# Supplementary material for: A Population Genomic Investigation of Immune Cell Diversity and Phagocytic Capacity in a Butterfly
Source: Genes (Basel). 2021 Feb 16;12(2):279. doi: 10.3390/genes12020279 (PMC7920040; doi:10.3390/genes12020279)
Supplement: Supplementary file 1 [file genes-12-00279-s001.pdf]

Table S1. Test Statistics for total hemocyte count by sex and population.

	Estimate	Std. Error	t value	Pr(>|t|)
(Intercept)	15716	1927	8.156	1.31e-09
PopulationSweden	-1853	2340	-0.792	0.434
SexMale	-1790	2350	-0.762	0.451

Table S2. Test Statistics for total number of Phagocytosing cells.

Fixed effects: Tot.no.of.cells ~ Population + Sex					
	**Value**	Std.Error	DF	t-value	p-value
(Intercept)	48.95381	6.345961	306	7.714168	0.0000
PopulationSpain	12.63784	7.328444	41	1.724492	0.0922
SexM	-19.04693	7.328444	41	-2.599042	0.0129

Table S3. Averages of proportion of phagocytic cell types by sex and population in percentages M= average, SD = standard deviation.

	Sweden				Spain			
	Female		Male		Female		Male	
	M	SD	M	SD	M	SD	M	SD
**Phagocytic granulo-cytes (%)**	89.05	6.51	81.83	5.91	88.92	3.61	84.76	11.97
**Phagocytic plasmato-cytes (%)**	3.21	2.15	6.52	6.32	3.82	4.29	7.83	8.48
**Phagocytic oenocytoids (%)**	7.75	5.07	11.66	4.05	7.27	2.48	7.39	4.04

Table S4. Test Statistics for proportion of Granulocytes between populations and sex.

Fixed effects: Prop_Gran ~ Population + Sex					
	**Value**	Std.Error	DF	t-value	p-value
(Intercept)	0.8500781	0.02208492	306	38.49134	0.0000
PopulationSpain	-0.0325426	0.02550832	41	-1.27576	0.2092
SexM	-0.0488211	0.02550832	41	-1.91393	0.0626

Table S5. Test Statistics for proportion of Plasmatocytes between populations and sex.

Fixed effects: Prop_Plasm ~ Population + Sex					
	**Value**	**Std.Error**	**DF**	**t-value**	**p-value**
(Intercept)	0.06973702	0.01936436	306	3.601307	0.0004
PopulationSpain	0.05291232	0.02236563	41	2.365787	0.0228
SexM	0.03345132	0.02236563	41	1.495657	0.1424

Table S6. Test Statistics for proportion of Oenocytoids between populations and sex.

Fixed effects: Prop_Oeno ~ Population * Sex					
	**Value**	**Std.Error**	**DF**	**t-value**	**p-value**
(Intercept)	0.06977273	0.01074684	306	6.492396	0.0000
PopulationSpain	0.00102273	0.01519832	40	0.067292	0.9467
SexM	0.03746586	0.01522313	40	2.461113	0.0183
Popula-tionSpain:SexM	-0.04371586	0.02151123	40	-2.032235	0.0488

Table S7. Test Statistics for total number of phagocytosing cells between population and sex.

Fixed effects: Tot.Phag.Tot.no.cells ~ Population + Sex					
	**Value**	**Std.Error**	**DF**	**t-value**	**p-value**
(Intercept)	0.7683284	0.02908282	306	26.418639	0.0000
PopulationSpain	-0.1338387	0.03358841	41	-3.984669	0.0003
SexM	0.0758705	0.03358841	41	2.258830	0.0293

Table S8. Test statistics for proportion of phagocytes between population and sex as measured by flowcytometry.

	**Estimate**	**Std.Error**	**tvalue**	**Pr(>|t|)**
(Intercept)	-0.28436	0.10548	-2.696	0.008040
PopulationSweden	0.49947	0.13242	3.772	0.000254
SexMale	0.01866	0.12947	0.144	0.885617

Table S9. Test Statistics for proportion of phagocytosing granulocytes.

Fixed effects: Prop_Phag_Gran ~ Population + Sex					
	**Value**	**Std.Error**	**DF**	**t-value**	**p-value**
(Intercept)	0.8814641	0.02014210	306	43.76228	0.0000
PopulationSpain	0.0151401	0.02326553	41	0.65075	0.5188
SexM	-0.0605946	0.02326553	41	-2.60448	0.0128

Table S10. Test Statistics for proportion of phagocytosing plasmatocytes.

Fixed effects: Prop_Phag_Plasm ~ Population + Sex					
	**Value**	**Std.Error**	**DF**	**t-value**	**p-value**
(Intercept)	0.03105434	0.01494720	306	2.0776024	0.0386
PopulationSpain	0.00914131	0.01726409	41	0.5294985	0.5993
SexM	0.03767687	0.01726409	41	2.1823837	0.0349

Table S11. Test Statistics for proportion of phagocytosing oenocytoids.

Fixed effects: Prop_Phag_Oeno ~ Population + Sex					
	**Value**	**Std.Error**	**DF**	**t-value**	**p-value**
(Intercept)	0.08854209	0.01096815	306	8.072658	0.0000
PopulationSpain	-0.02447054	0.01267218	41	-1.931044	0.0604
SexM	0.02231145	0.01267218	41	1.760663	0.0858

Table S12. Test Statistics for propensity of granulocytes to perform phagocytosis.

	Estimate	Std. Error	t value	Pr(>|t|)
(Intercept)	1.3910	0.1704	8.162	3.98e-10
PopulationSpain	-0.6058	0.1953	-3.103	0.00347
SexM	0.4533	0.1940	2.337	0.02442

Table S13. Test Statistics for propensity of plasmatocytes to perform phagocytosis.

	Estimate	Std. Error	t value	Pr(>|t|)
(Intercept)	-0.4672	0.2461	-1.899	0.0647
PopulationSpain	-0.9893	0.2963	-3.339	0.0018
SexM	0.7188	0.2949	2.437	0.0192

Table S14. Test Statistics for propensity of oenocytoids to perform phagocytosis.

	Estimate	Std. Error	t value	Pr(>|t|)
(Intercept)	1.7119	0.2341	7.313	5.97e-09
PopulationSpain	-1.0185	0.2680	-3.801	0.000471
SexM	0.8344	0.2633	3.169	0.002887

Table S15. Distribution of F_{ST} values G stands for genome-wise values, whereas P is the phagocytosis genes values.

	**SNPs**				**Gene level**			
**Percentile**	**F_{ST} within genes**		**F_{ST} 5kb genes**		**F_{ST} within genes**		**F_{ST} 5kb genes**	
	**G**	**P**	**G**	**P**	**G**	**P**	**G**	**P**
**100%**	1	0,308	1	0.714	0.795	0.142	0.638	0.079
**99.5%**	0.329	0,256	0.350	0.273	0.268	0.142	0.201	0.079
**97.5%**	0.191	0,191	0.204	0.178	0.141	0.097	0.118	0.064
**75%**	0.065	0,068	0.065	0.063	0.059	0.060	0.063	0.054
**50%**	0.034	0,032	0.033	0.033	0.043	0.041	0.053	0.049
**2.5%**	0.002	0,003	0.002	0.003	0	0	0.033	0.036
**0%**	0	0,001	0	0	0	0	0	0.036
**Average**	0.049	0,049	0.050	0.047	0.049	0,038	0.058	0.049
**Std Dev**	0.057	0,049	0.059	0.049	0.042	0,027	0.026	0.007

Table S16. The significant GO terms with $p < 0.01$ of the SNPs with the highest 2.5 % values (exons only).

**GO_term**		**Annotated**	**Significant**	**Expected**	**classicFisher**
**GO:1901135**	carbohydrate derivative metabolic process	159	36	21.50	0.00092
GO:0006471	protein ADP-ribosylation	3	3	0.41	0.00246
GO:1901136	carbohydrate derivative catabolic proces	9	5	1.22	0.00350
GO:0000270	peptidoglycan metabolic process	6	4	0.81	0.00396
GO:0006027	glycosaminoglycan catabolic process	6	4	0.81	0.00396

GO:0009253	peptidoglycan catabolic process	6	4	0.81	0.00396
GO:0044710	single-organism metabolic process	781	129	105.63	0.00404
GO:0005975	carbohydrate metabolic process	176	36	23.80	0.00585
GO:0046434	organophosphate catabolic process	10	5	1.35	0.00622
GO:0006026	aminoglycan catabolic process	7	4	0.95	0.00825
GO:0009166	nucleotide catabolic process	7	4	0.95	0.00825
GO:1901292	nucleoside phosphate catabolic process	7	4	0.95	0.00825

Table S17. The GO terms with terms with p < 0.01 of the SNPs with the highest 2.5 % FST values (with 5kb both sides).

GO_term		Annotated	Significant	Expected	classicFisher
GO:0006508	proteolysis	380	45	14.90	1.6e-12
GO:0008152	metabolic process	2436	119	95.54	8.4e-06
GO:0006542	glutamine biosynthetic process	5	3	0.20	0.00056
GO:0044710	single-organism metabolic process	781	46	30.63	0.00154
GO:0019538	protein metabolic process	872	50	34.20	0.00162
GO:0006541	glutamine metabolic process	7	3	0.27	0.00184
GO:0055114	oxidation-reduction process	391	26	15.34	0.00434
GO:0008652	cellular amino acid biosynthetic process	19	4	0.75	0.00555
GO:1901607	alpha-amino acid biosynthetic process	19	4	0.75	0.00555
GO:0009084	glutamine family amino acid biosynthetic...	11	3	0.43	0.00773
GO:0009064	glutamine family amino acid metabolic pr...	13	3	0.51	0.01265
GO:0016053	organic acid biosynthetic process	28	4	1.10	0.02233
GO:0046394	carboxylic acid biosynthetic process	28	4	1.10	0.02233
GO:0006265	DNA topological change	7	2	0.27	0.02818
GO:1901605	alpha-amino acid metabolic process	33	4	1.29	0.03841

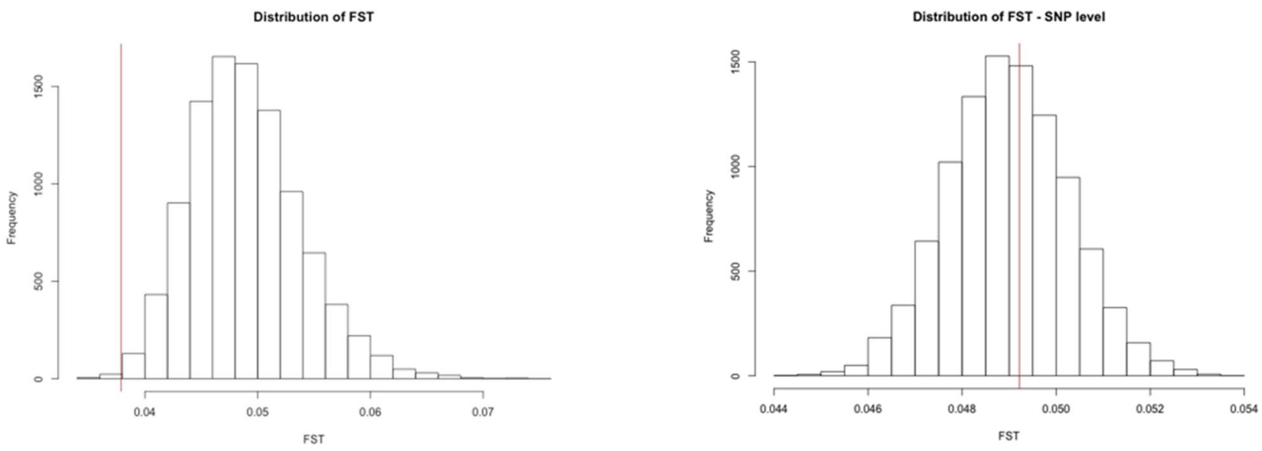

(a) (b)

Figure 1. Distribution plots of the permutations for genome-wide FST, left (a) calculated per gene, right (b) per SNP. The red line denotes the averages of these values for the phagocytosis genes.
